# Supplementary material for: A survey of UK beekeeper’s Varroa treatment habits
Source: PLoS One. 2023 Feb 15;18(2):e0281130. doi: 10.1371/journal.pone.0281130 (PMC9931098; doi:10.1371/journal.pone.0281130)
Supplement: S1 Fig — The data was compiled from the annually published data contained in the BBKA newsletter. We have added in the 5-year rolling average. (DOCX) [file pone.0281130.s001.docx]

Supplementary Data

A survey of UK Beekeeper’s treatment habits

Alexandra Valentine*, Stephen J. Martin

School of Science, Engineering and Environment, The University of Salford, M5 4WT, Manchester, UK

*Figure S1*: The annual overwintering colony losses in England and Wales recorded by the BBKA survey conducted by Dr D. Aston. The data was compiled from the annually published data contained in the BBKA newsletter. We have added in the 5-year rolling average.
