# Supplementary material for: A survey of UK beekeeper’s Varroa treatment habits
Source: PLoS One. 2023 Feb 15;18(2):e0281130. doi: 10.1371/journal.pone.0281130 (PMC9931098; doi:10.1371/journal.pone.0281130)
Supplement: S2 Fig — The university logo has been implemented to increase the credibility of the survey. (DOCX) [file pone.0281130.s002.docx]

*Figure S2*: A copy of the online survey as was seen by participants. The university logo has been implemented to increase the credibility of the survey.
