# Supplementary material for: A survey of UK beekeeper’s Varroa treatment habits
Source: PLoS One. 2023 Feb 15;18(2):e0281130. doi: 10.1371/journal.pone.0281130 (PMC9931098; doi:10.1371/journal.pone.0281130)
Supplement: Attachment — Submitted filename: PONE-D-22-05221_R1 Survey paper rev.pdf [file pone.0281130.s004.pdf]

PLOS ONE

A survey of UK Beekeeper’s Varroa treatment habits

--Manuscript Draft--

**Manuscript Number:**	PONE-D-22-05221R1
**Article Type:**	Research Article
**Full Title:**	A survey of UK Beekeeper’s Varroa treatment habits
**Short Title:**	UK Beekeeper’s Varroa treatment habits
**Corresponding Author:**	Stephen J. Martin<br>University of Salford<br>Sheffield, UNITED KINGDOM
**Keywords:**	Varroa destructor; Survey; beekeepers; UK; Varroa treatment; treatment-free beekeeping:resistance
**Abstract:**	The global spread of the parasitic mite Varroa destructor instigated a substantial decline in both managed and feral honeybee (Apis mellifera) colonies mainly across the Northern hemisphere. In response, many beekeepers began to treat their colonies with chemical acaricides to control mite populations in managed colonies. However, some countries or beekeepers allowed their bees to develop mite-resistance by adopting a “treatment-free” approach, rather than using selective breeding programs. Yet, the distribution and proportion of beekeepers either treating or not within the United Kingdom (UK) is unknown, as it is in most Northern hemisphere countries. Therefore, the aim of this study was to conduct a beekeeper survey to determine the current treatment strategies within the UK. We gathered 2,872 beekeeper responses from an estimated 30,000 UK beekeepers belonging to 242 bee-associations in the winter of 2020/21. The survey indicated that the majority (72-79%) of UK beekeepers are still treating their bees for Varroa , typically twice-yearly using chemical-based methods. Despite this, an estimated 3,000 UK beekeepers are treatment-free for over six years or have never treated. This is reflected by our finding that only 78 associations out of 242 consist of responders who entirely treated, whereas only four associations had more than 75% of their members that were treatment-free. Although overall treatment status was not affected by association currently, we predict that in time the shift towards intervention-free approaches could lead to increasing numbers of Varroa -resistant managed UK honeybee colonies.
**Order of Authors:**	Stephen J. Martin
	Alexandra Valentine
**Response to Reviewers:**	Data is said to be publicly available, but no link included. Same goes for the supplemental information.<br>THE DATA WILL BE PLACED ON DRYAD IF ACCEPTED<br>Please apply the use of commas for numbers consistently. For example, compare line 35 and line 38. Also Varroa is not consistently italicized. DONE<br>I don’t understand why the feral population of bees is mentioned in line 43. You surveyed beekeepers with managed colonies. This has no implications for the feral bees and no evidence is presented that the UK has truly feral colonies.<br>DELETED ‘FERAL COLONIES’ AS THE IDEA WAS THAT MORE VARROA-RESISTANT MANAGED COLONIES WILL LEAD TO MORE VARROA-RESISTANT FERAL COLONIES BUT WE AGREE WITH THE COMMENT TO DELETED.<br>Line 56-57, awkward sentence structure. Unclear what remained high. ENTIRE SECTION REWORDED<br>Line 60, based used twice REPLACED ONE WITH ESTABLISHED<br>Line 65-66, not clear why an IPM strategy would be linked to long-term resistance and low mite levels. IPM, as I understand it, is threshold base and involves monitoring and then treating appropriately. How does this encourage low mite levels? And why would this lead to long-term resistance? Needs explanation and evidence.<br>THIS IS JUST A REMOTE POSSIBILTY SO THIS STATEMENT HAS BEEN DELETED<br>Line 102: I have no access to the Figure S1<br>Line 120: I have no access to Table S1 SORRY THIS HAS OCCURRED

Powered by Editorial Manager® and ProduXion Manager® from Aries Systems Corporation

	Line 201-203, that assumes that the survey reached and was completed by a representative wedge of the beekeeping population. Many treatment-free beekeepers tend to be younger and more active online, so this may have skewed the responses. These caveats should be mentioned.<br>THE COMMENT ABOVE ONLINE ACTIVE HAS NOW BEEN ADDED TO THE RELEVENT PART OF THE DISCUSSION L 239-241.<br>Line 218-219: This is not surprising at all. Most treatment free beekeepers in the United States expect annual losses of 50%. Thus, in order to rebuild the following year, they must keep more colonies in reserve. I am disappointed this survey didn't ask for self-reporting of annual loss rates or honey production per colony. The high level of treatment-free suggests that beekeepers and the much higher colony numbers suggests that the resistance is probably not enough for low annual losses.<br>WE DID NOT INCLUDE COLONY DEATHS IN OUR SURVEY SINCE THIS IS ALREADY COVERED BY THE LONG RUNNING BBKA SURVEY DATA FROM WHICH WE HAVE NOW INCULDED(see Fig S2) AND WE HAVE EXPANDED THE PARAGRAPHY COMPAIRING COLONY LOSS RATES FROM UK TREATMENT-FREE BEEKEEPERS. MY PERSONAL EXPERINCE WORKING WORK WITH SEVERAL TREATMENT-FREE BEEKEEPERS THEIR LOSSES ARE SIMILAR TO BEEEKEEPERS THAT ARE TREATING.<br>WE DID NOT ASK ABOUT HONEY PRODUCTION AS IN THE UK THIS VARIES GREATLY DUE TO LOCATION AND MICRO-CLIMATE. CLEARLY THERE ARE BIG DIFFERENCES BETWEEN THE SITUATION IN THE UK AND USA IN NUMBER OF TREATMENT FREE BEEKEEPERS THIS IS NOW MENTION IN THE LAST SECTION AS WELL AS THE (Thoms et al)<br><br>Line 235-240: This makes the assumption that IPM requires multiple treatments per year. IPM requires monitoring and treating accordingly when a pest level rises above a certain point. Also, many do not consider drone trapping a treatment. As I don't have an actual copy of the survey questions, I cannot assess if there may have been misunderstandings in the responses.<br>POOR PLACEMENT OF SENTENCE SO RE-STRUCTED THIS SECTION AND COMMENTED THAT ONLY VERY BASIC MITE MONTIROING TAKES PLACE.<br>Line 253-255: This needs more clarification. Is the 4% what beekeepers reported was specifically due to varroa? This would then be an underrepresentation of actual winter losses due to varroa. Numerous surveys have shown that when varroa levels are high, winter losses increase, even though beekeepers blame other factors for the loss. Many beekeepers with colony winter losses don't know what killed their colony, but beekeepers with more experience can often diagnose the contracted brood nest area and varroa frass.<br>WE HAVE GIVEN MORE CLARIFICATION ON THE PRINCIPAL CAUSES OF COLONY LOSSES IN THE UK<br>Line 254-255: Why is the low winter loss attributed to treatment-free beekeeping. There could be other explanations, such as very active beekeeping groups that teach how to monitor and treat effectively. The evidence presented does not warrant this statement, as there is no segregation of losses by those treating and those not treating presented.<br>WE AGREE THAT NOT ENOUGH EVIDENCE IS PRESENT AT THE MOMENT SO THE SENTENCE HAS BEEN DELETED<br>Line 256-260: These statements require the reader to go to the original articles and assess the numbers of colonies surveyed. Important information such as colonies assessed in the survey should be included with the paraphrased results, especially as these are not peer-reviewed studies but association results and so the statistics have not been evaluated. The way they are presented in this paper, the reader assumes they are peer-reviewed papers, unless the references are checked in detail.<br>WE AGREE SO ADDED IN ADDITIONAL DATA/INFO BOTH IN THE TEXT AND SUPP DATA<br>Line 276-279: This is a very broad statement and is not justified by the results presented, as no losses were calculated in the survey.<br>WE HAVE SHOWEN USING BBKA SURVEY BASED ON 2,613 BEEKEEPERS THAT THERE IS NO SIG RELATIONSHIP BETWEEN %TREATMENT AND %COLONY LOSSES AT A REGIONAL LEVEL IN THE UK. WE HAVE ALSO ADDED IN MORE REFRENCES TO SUPPORT OUR BROAD STATEMENT.<br>FINALLY THE PAPER HAS BEEN CAREFULLY PROOFED NOW<br><br>5. Review Comments to the Author

Powered by Editorial Manager® and ProduXion Manager® from Aries Systems Corporation

	Reviewer #1: The submitted survey of UK beekeeper´s varroa treatment habits presents interesting new data and fills a gap in our knowledge on recent beekeeping practice. The study is well presented with a clear description of the data sampling and evaluation procedure. The discussion picks up relevant links to related surveys and the establishment of mite resistance in bee populations depending on the treatment strategies. The length of the different chapters is appropriate and well balanced.<br>Some confusion may arise about the number of respondents for the data in table 3. The table reports on a total of 2,868 responses from 2,897 responses in general (L116). If 25 responses from the Isle of Man have been excluded, some of the 16 participants with zero colonies (L146f) seem to be included here. However, this wouldn´t make any sense for my understanding. The authors should give a precise description on this.<br>THE 25 RESPONSES FROM THE ISLE OF MAN WERE EXCLUDED FROM THE 2897 (= 2872) WHICH IS NOW STATED IN THE METHODS. IN TABLE 1 THE 16 BEEKEEPERS WITH ZERO COLONIES WHERE EXCLUDED WHICH LEAVES 2856 BEEKEEPERS AS STATED IN TABLE 1. WE HAVE REWRITTEN THE METODS TO INCLUDE ALL SAMPLES EXCULDED<br>L102 refers to Fig S1 which is missing in the supplied manuscript. (Sorry our error, CORRECTED)<br>In L120 Table S1 is mentioned but it should be Table 5 as far as I understand. (deleted)<br>In L 264 the second “by” has to be erased. Deleted<br>Reviewer #2: The paper has the potential to be interesting but is currently missing the access to the data and the supplemental information, so difficult to assess. Also, the conclusions are very broad, and the data presented don't support the conclusions the authors make, as the survey did not include any assessment of colony losses. Without distinguishing what the actual loss rates of the treatment free beekeepers have, it is not possible to state that the bees are resistant to varroa or have a high tendency for survival. The higher colony numbers actual suggests the opposite.<br>AS MENTIONED ABOVE WE HAVE A PARAGRAPH COMPARING LOSSES IN TREATMENT-FREE POPULATIONS VS THE NATIONAL AVERAGE. ANOTHER SURVEY COLLECTS LOSS DATA BUT NOT AIMED AT SEPARATING OUT THE TREATED AND NOT TREATED. WE HAVE ALSO INCLUDED THE MAIN REASONS IN ENGLAND & WALES FOR COLONY LOSSES AND VARROA ACCOUNTS FOR ONLY 4% ALTOUGH WE AGRREE THIS MAY BEEN UNDERESTIMATED BY THE BEEKEEPERS.<br><br>Also there are a number of odd sentence structures and grammatical errors. I have made note of the mores specific comments in a separate document. ALL SHOULD BE FIXED NOW<br>Reviewer #3: The study aims to estimate the number of beekeepers in the UK who are not treating their colonies for varroa, and to estimate whether the existence of untreated and resistant colonies in the UK could be assumed. Information on beekeeping practices is currently limited in the scientific literature, and this question and the data the study provides are relevant and valuable. The article is generally clear and well written. The methodological choices are globally sound and with a high number of respondents to the survey, which is important to note. Still some information should be added in the methodological section, and the limits induced by some methodological choices should be identified and discussed as they can have important impacts on the interpretation of the results. Also, the findings should be discussed regarding the scientific literature on the subject. For these reasons I recommend major revisions.<br><br><br>Major comments<br>Some methodological limits should be identified and discussed, especially i) the possible influence of the sampling through the beekeeping associations (are these associations involved in prescribing varroa treatments?)<br>THERE IS NO PRESCRIPTION OF TREATMENTS VIA ASSOCIATIONS SINCE TREATMENT ADVICE COMES CENTRALLY FROM THE NATIONAL BEE UNIT & THE MAP INDICATES WE HAD A VERY WIDE SPREAD OF RESPONSE<br>and ii) the lack of information that the survey provides about the colony losses and about the beginning year of the beekeeping activity. Without this last information, the share of beekeepers who have “never treated” cannot be interpreted as it is done

currently regarding the possible existence of surviving colonies (those beekeepers could have started recently, and their colonies may not have faced this absence of treatment for varroa for a long time). It should not be suggested that these colonies had never known any treatment (it could be the case, but the data of the survey do not allow to conclude on this question).
THERE ARE A GROWING GROUP OF BEEEKEEPERS IN THE UK THAT NEVER TREAT SUCH AS OXFORDSHIRE NATURAL BEEKEEPING ASSOCIATION AND THEIR LOSSES FOR TWO WINTERS WERE SIMILAR TO THE NATIONAL AVERAGE. THIS INFORMATION IS PROVIDED IN THE MS AS WELL AS A NEW SUPPLEMENTAL FIGURE SHOWING THE NATIONAL AVERAGE COLONY LOSSES FROM 2007 TO 2021.

The existence of colonies surviving without treatment for varroa and the possible losses that the absence of treatment may induce should also be discussed regarding the scientific literature on these questions.
WE HAVE EXPANDED THE SECTION ON COLONY LOSSESS IN THE UK AS REQUESTED

Other comments
L. 57: “due to almost universal treatment by beekeepers”: this statement should be either nuanced or supported by a reference about American beekeepers’ practices. E.g. see Thoms et al. (2019 - https://doi.org/10.1007/s13280-018-1130-z), which indeed reveals a high percentage of American backyard beekeepers reporting not to treat for varroa.
I CHECKED THOMS ET AL AND ALOUGHT MOST TREATMENT SKEPICS DO NOT TREAT THIS GROUP MADE UP LESS THAN 3% OF THE SURVEY EG (63 VS 2276). THUS, IT APPEARS THAT THERE ARE MUCH FEWER TREATMENT-FREE BEEKEEPERS IN THE USA THAN UK. HOWEVER, WE HAVE CHANGED ‘UNIVERSAL’ TO ‘HIGH’ WHICH IS MORE ACCURATE AND CITED THIS PAPER AT THE END OF THE DISCUSSION.

L. 100-105: If I understand correctly, there was no question about the colony survival (cf. L100-105). It would have been a valuable information, as the emergence of resistant colonies is only possible if the non-treated colonies survive (in the case of backyard beekeepers replacing regularly their colonies that died from varroa, these non-treated colonies would not be more resistant than other).
SEE EALIER COMMENTS ABOUT COLONY LOSSES

L. 106-113. As the beekeeping associations who forward the survey to their adherents play a major role in the sampling, it would be useful to provide more information about these associations (as such beekeeper associations and their role can differ from one association or one country to the other). Especially, can these associations interfere with their adherents’ treatment choices (e.g., by advising or not advising to treat for varroa)?
THIS IS SOMETHING THAT IS NOT DONE THE ASSOICATIONS ONLY FORWARED THE SURVEY LINK TO ALL ITS MEMBERS USING THERE (CONFEDENTIAL DATABASE). IF THESE TYPE OF BEHAVIOR DID OCCUR, WE WOULD HAVE PICKED IT UP IN THE ANAYLSIS. FURTHERMORE, THE ASSOICATIONS THAT SEND OUT THE LINK HAVE NO WAY OF SEEING WHAT THEIR MEMBERS WORTE.

L. 123. One of the main analysis choices is the investigation for a possible spatial pattern. As many other factors could possibly influence beekeepers’ choice about their treatment for varroa (beekeeping experience, age, etc), this hypothesis of a spatial pattern could be explained and justified.
EACH ASSOICATION HAS A WIDE RANGE OF AGES, GENDERS, AND EXPERIENCE TO REQUEST THIS TYPE OF DATA AND CONSDIER THEM IN THE ANAYLSIS LIES WAY BEYOND THE SCOPE OF THIS STUDY.

L. 130. Some biomechanical and natural methods that were reported by beekeepers (cf. Table 5) are not efficient against varroa. As the study focuses on the possible existence of untreated / resistant colonies and not on the choice of beekeepers to treat or not to treat (reasons for such choices, etc), these “low-efficacy” treatments could have been considered jointly with the treatment-free group, or a third variant for the

Powered by Editorial Manager® and ProduXion Manager® from Aries Systems Corporation

response variable could have been considered. So, the choice to gather all the treatments together in a single response variable could be explained.
THE REVIEWER IS CORRECT IN THAT THE DIFFERENT TREATMENTS RANGE WIDLY IN THEIR EFFICACY'S. TO ERR ON THE SIDE OF CAUTION WE HAD TO MAKE IT VERY CLEAR TO UNDERSTAND WHAT 'TREATMENT-FREE' MEANT. OTHERWISE, WE COULD HAVE RUN THE PROBLEM OF BEEKEEPERS DECIDING WHAT THEY CONSIDER IS TREATMENT FREE. WE AGREE THE LOW-EFFICACY TREEATMENTS COULD BEE ALLOWING NATURAL RESISTANT TO DEVELOP AND THIS ONE OPTION FOR BEEKEEPERS TO BECOME TREATMENT FREE. IN THE FUTURE IT WOULD BE INTRESTING TO SEE IF THE USAGE OF THESE LOW-EFFICACY METHODS INCREASED.

L. 133. As one of the questions of the study is the existence of untreated and possibly resistant colonies, it would be useful to know how many colonies in total these 2,872 beekeepers manage and what percentage of the total number of colonies in the UK it represents.

WE HAVE NOW ADDED IN A SECTION AT THE START OF THE RESULTS SHOWING THAT SURVEY ALSO COVERED ABOUT 10% OF THE UK COLONIES

L. 141-144. This passage is not very clear, especially what is supposed to "explained why there was significant increase in treatment-free beekeepers as the numbers of colonies they managed increased". It would be useful to rephrase or to complete it.
THIS SECTION HAS BEEEN REEWORDED

L. 146 / Table 1. The third column heading is not very clear: does it represent the percentage of colonies of the group (1-5,…) which are not treated? So, 73% of the colonies of the 16-30 group are not treated?
An additional column providing the percentage of treatment-free beekeepers in each of the groups would be welcome. Besides, the final percentage of untreated colonies (gathering all groups) should be added.

WE RE-CHECKING THE DATA IN TABLE 1 WE DISCOVER AN ERROR SO THANKS FOR THAT. WE HAVE NOW RE-DONE TABLE 1 AND TABLE 2 SO SHOULD BE MUCH CLEARER AS REQUESTED BY THE REVIEWER
SORRY ITS BEEEKEEPERS NOT COLONIES SO WE HAVE REWORDED IT.

L. 160-162. Grouping the beekeepers who indicated that they have "never treated" with those indicating a specific number of years since the last treatment (here 10 years and more, or 6 years and more L. 201) is questionable as the date of installation of the beekeepers was not in the survey and is not known. Some of them could have started beekeeping recently, and the fact that they have "never treated" for varroa does not presume that their colonies had to face a significant period without treatment. This group of beekeepers with an unknown number of years without treatment should be considered separately and the confusion with the groups where a long treatment-free period is known should be avoided.

IN TABLE 2 WE HAVE SEPRATED OUT THE NEVER ADMINISTRED GROUP SO THEY ARE CONSIDER SEPRATLY. THE MANY OF THESE BEEKEEPERS BELONG TO THE OXFORD NATURAL BEEKEEPING. THIS GROUP HAS BEEN AROUND FOR 10 YEARS AND WE PROVIDE COLONY LOSS DATA FROM THIS GROUP AND COMPARE AGAINST THE NATIONAL AVERGAE AND ITS VERY SIMILAR.

L. 163-164. The fact than some association gather more than 75% of treatment-free beekeepers raises questions about the role of these associations, and about the possible exchanges related to varroa treatments that its members may have. Even if it was not the objective of the study to understand the determinants of the absence of treatment, it would be interesting to discuss the possible role of associations on this point given their central place in the survey sample.

AS PREVIOUSLY MENTIONED VARROA TREATMEENTS ARE RECOMMENDED BY THE GOVERMENTS NATIONAL BEE UNIT. THE TREATMENT-FREE BEEKEEPERS IN MOST ASSOICATIONS DO NOT MENTION THAT THEY DO NOT TREAT AS THEY ARE IN MINORITY HENCE WHY WE DID THE SURVEY TO GET A

Powered by Editorial Manager® and ProduXion Manager® from Aries Systems Corporation

	BETTER ESITATMTION OF THEE THE NUMBER OF TREATMENT FREE BEEKEEPERS.<br><br>L. 178. “the majority are treated”: the majority of colonies? YES, SO CHANGED<br><br>L. 180-184. It would be useful to add the global percentage of beekeepers not using any treatment and to more clearly distinguish if the percentages given for the types of treatments are exclusive of each other or not. E.g., can the 3% of beekeepers using biomechanical methods be also in the 78% using chemical? Or are they only using biomechanical methods?<br><br>THIS DATA DOES NOT CURRENTLY EXIST THE Thoms et al survey and this study are some of the first attempts to collect such data.<br><br>L. 201. See comment on L. 160-162<br><br>L. 202. The extrapolation of the study results to the 30,000 estimated UK beekeepers should be supported by a discussion about the representativity of the sample. That joins the question of giving information about the beekeeping associations (see comment on L 103-115), and about their representativeness (are they some apicultural practices which may differ between members of these associations and beekeepers who are not members, e.g., if the associations are involved in prescribing treatments for varroa? Are the members of these associations representing a large share of the UK beekeepers?)<br>IN THE UK THERE ARE AROUND 30,000 AMATEUR BEEKEEPERS BUT ONLY 400 PROFESSIONALS THAT BELONG TO THE BEE FAMERS SOCIETY. THIS IS NOW MENTIONED IN THE METHODS.<br><br>L. 257-260. As varroa is usually considered in scientific and technical literature as an important factor of colony losses, some scientific references about the place of varroa in colony losses need to be added and the technical references which are the only provided references should be discussed regarding these scientific references.<br><br>THIS IS VERY TRUE PARTICULAR IN THE NORTHERN HEMISPHERE BUT IN MANY COUNTRIES (E.G., AFRICA, BRAZIL-MEXICO, CUBA) VARROA IS NOW NOT CONSIDERED A PROBLEM SINCE THE BEES ARE ABLE TO KEEP THE INFESTATION LEVEL DOWN BELOW 10-4% DEPENDING ON THE POPULATION. HENCE IN THE EYES OF WELL-ESTABLISHED (6YRS+) TREATMENT-FREE BEEKEEPERS VARROA IS NOT CONSIDERED AS A SERIOUS PROBLEM. THE SAME CAN BE SAID BY BEEKEEPERS THAT REGULAR TREAT THEIR VARROA POPULATIONS. WE HAVE NOW ADDED IN A SHORT SECTION SHOWING IN ONE SURVEY THE BEEKEEPERS REPORTED THAT ONLY 4% OF THEIR DEATHS WAS DIRECTLY CAUSED BY VARROA.<br><br>L. 267. It would be useful to precise “in Hawaii” and not only “in the USA” as the context of an island can be specific. CHANGED AS SUGGESTED<br><br>Fi. 1. A. A legend with the number of respondents corresponding to the different pie sizes is needed here. THIS HAS NOW BEEN DONE
**Additional Information:**	
**Question**	**Response**
**Financial Disclosure**<br><br>Enter a financial disclosure statement that describes the sources of funding for the work included in this submission. Review the submission guidelines for detailed requirements. View published research articles from *PLOS ONE* for specific examples.	The authors received no specific funding for his work.

Powered by Editorial Manager® and ProduXion Manager® from Aries Systems Corporation

This statement is required for submission and **will appear in the published article** if the submission is accepted. Please make sure it is accurate.<br><br>**Unfunded studies**<br>Enter: *The author(s) received no specific funding for this work.*<br><br>**Funded studies**<br>Enter a statement with the following details:<br>• Initials of the authors who received each award<br>• Grant numbers awarded to each author<br>• The full name of each funder<br>• URL of each funder website<br>• Did the sponsors or funders play any role in the study design, data collection and analysis, decision to publish, or preparation of the manuscript?<br>• **NO** - Include this sentence at the end of your statement: *The funders had no role in study design, data collection and analysis, decision to publish, or preparation of the manuscript.*<br>• **YES** - Specify the role(s) played.<br><br>* typeset	
**Competing Interests**<br><br>Use the instructions below to enter a competing interest statement for this submission. On behalf of all authors, disclose any competing interests that could be perceived to bias this work—acknowledging all financial support and any other relevant financial or non-financial competing interests.<br><br>This statement is required for submission and **will appear in the published article** if the submission is accepted. Please make sure it is accurate and that any funding sources listed in your Funding Information later in the submission form are also declared in your Financial Disclosure statement.<br><br>View published research articles from	The authors have declared that no competing interests exist.

PLOS ONE for specific examples.<br><br>**NO authors have competing interests**<br><br>Enter: *The authors have declared that no competing interests exist.*<br><br>**Authors with competing interests**<br><br>Enter competing interest details beginning with this statement:<br><br>*I have read the journal's policy and the authors of this manuscript have the following competing interests: [insert competing interests here]*<br><br>* typeset	
**Ethics Statement**<br><br>Enter an ethics statement for this submission. This statement is required if the study involved:<br><br>• Human participants<br>• Human specimens or tissue<br>• Vertebrate animals or cephalopods<br>• Vertebrate embryos or tissues<br>• Field research<br><br>Write "N/A" if the submission does not require an ethics statement.<br><br>General guidance is provided below. Consult the submission guidelines for detailed instructions. **Make sure that all information entered here is included in the Methods section of the manuscript.**	Ethical clearance was given by the University of Salford, UK ethics committee. All data were analyzed anonymously.

Powered by Editorial Manager® and ProduXion Manager® from Aries Systems Corporation

**Format for specific study types**<br><br>**Human Subject Research (involving human participants and/or tissue)**<br>• Give the name of the institutional review board or ethics committee that approved the study<br>• Include the approval number and/or a statement indicating approval of this research<br>• Indicate the form of consent obtained (written/oral) or the reason that consent was not obtained (e.g. the data were analyzed anonymously)<br><br>**Animal Research (involving vertebrate animals, embryos or tissues)**<br>• Provide the name of the Institutional Animal Care and Use Committee (IACUC) or other relevant ethics board that reviewed the study protocol, and indicate whether they approved this research or granted a formal waiver of ethical approval<br>• Include an approval number if one was obtained<br>• If the study involved *non-human primates*, add *additional details* about animal welfare and steps taken to ameliorate suffering<br>• If anesthesia, euthanasia, or any kind of animal sacrifice is part of the study, include briefly which substances and/or methods were applied<br><br>**Field Research**<br><br>Include the following details if this study involves the collection of plant, animal, or other materials from a natural setting:<br>• Field permit number<br>• Name of the institution or relevant body that granted permission	
**Data Availability**<br><br>Authors are required to make all data underlying the findings described fully available, without restriction, and from the time of publication. PLOS allows rare exceptions to address legal and ethical concerns. See the PLOS Data Policy and FAQ for detailed information.	Yes - all data are fully available without restriction

A Data Availability Statement describing where the data can be found is required at submission. Your answers to this question constitute the Data Availability Statement and **will be published in the article**, if accepted.<br><br>**Important:** Stating 'data available on request from the author' is not sufficient. If your data are only available upon request, select 'No' for the first question and explain your exceptional situation in the text box.<br><br>Do the authors confirm that all data underlying the findings described in their manuscript are fully available without restriction?	
**Describe where the data may be found in full sentences. If you are copying our sample text, replace any instances of XXX with the appropriate details.**<br><br>• If the data are **held or will be held in a public repository**, include URLs, accession numbers or DOIs. If this information will only be available after acceptance, indicate this by ticking the box below. For example: *All XXX files are available from the XXX database (accession number(s) XXX, XXX.*).<br>• If the data are all contained **within the manuscript and/or Supporting Information files**, enter the following: *All relevant data are within the manuscript and its Supporting Information files.*<br>• If neither of these applies but you are able to provide **details of access elsewhere**, with or without limitations, please do so. For example:<br><br>*Data cannot be shared publicly because of [XXX]. Data are available from the XXX Institutional Data Access / Ethics Committee (contact via XXX) for researchers who meet the criteria for access to confidential data.*<br><br>*The data underlying the results presented in the study are available from (include the name of the third party*	All relevant data are within the manuscript and its Supporting Information files.

and contact information or URL).<br>• This text is appropriate if the data are owned by a third party and authors do not have permission to share the data.<br><br>* typeset	
Additional data availability information:	

Manuscript

Click here to access/download;Manuscript;Survey paper V5 PlosONE REVISED9AV.docx

A survey of UK Beekeeper's *Varroa* treatment habits

Alexandra Valentine[1,2] and Stephen John Martin[1]*

1 School of Science, Engineering and Environment, The University of Salford, M5 4WT, Manchester, UK

2 Current address *School of Natural Sciences, National University of Ireland Galway, Ireland*

a.valentine2@nuigalway.ie

*** Correspondence to; s.j.martin@salford.ac.uk** orcid.org/0000-0001-9418-053X

Abstract

The global spread of the parasitic mite *Varroa destructor* instigated a substantial decline in both managed and feral honeybee (*Apis mellifera)* colonies mainly across the Northern hemisphere. In response, many beekeepers began to treat their colonies with chemical acaricides to control mite populations in managed colonies. However, some countries or beekeepers allowed their bees to develop mite-resistance by adopting a "treatment-free" approach, rather than using selective breeding programs. Yet, the distribution and proportion of beekeepers either treating or not within the United Kingdom (UK) is unknown, as it is in most Northern hemisphere countries. Therefore, the aim of this study was to conduct a beekeeper survey to determine the current treatment strategies within the UK. We gathered 2,872 beekeeper responses from an estimated 30,000 UK beekeepers belonging to 242 bee-associations in the winter of 2020/21. The survey indicated that the majority (72-79%) of UK beekeepers are still treating their bees for *Varroa*, typically twice-yearly using chemical-based methods. Despite this, an estimated 3,000 UK beekeepers are treatment-free for over six years or have never treated. This is reflected by our finding that only 78 associations out of 242 consist of responders who entirely treated, whereas only four associations had more than 75% of their members that were treatment-free. Although overall treatment status was not affected by association currently, we predict that in time the shift towards intervention-free approaches could lead to increasing numbers of *Varroa*-resistant managed UK honeybee colonies.

Introduction

Honeybees are key pollinators of crops and wildflowers [1]. They contribute globally more than €53 billion annually to the economy [2]. Still, honeybees are presently facing multiple stressors such as habitat destruction, pests, and pathogens. Across the Northern hemisphere the spread of the ecto-parasitic mite *Varroa destructor* (*Varroa*) and the key viral pathogen it transmits (Deformed Wing Virus) is the number one threat faced by beekeepers over past decades, causing an increase in over-wintering losses [3, 4]. In the UK, this was evidenced in the very high losses of managed colonies and the near eradication of feral colonies within a few years of *Varroa* becoming established [5]. Although, over-wintering colony losses have been declining in the UK during the past decade's (Fig. 1S) since *Varroa*-treatments became widespread [6]. Although colony losses remain consistently high in the USA [7], which is unexpected due to the high levels of treatment carried out by beekeepers to control *Varroa* populations. Typically, a variety of highly effective acaricides are primarily used to control *Varroa* populations, but with the development of acaricide resistance [8] alternative commercially based treatments established around Thymol and organic acids (Oxalic and Formic) have become increasingly popular. More time consuming and often less effective biomechanical treatment methods can also be used, such as powdered sugar and drone trapping. The application of Integrated Pest Management (IPM) techniques is suggested to be the most effective way [9], to treat mites since it involves a variety of methods applied in rotation throughout the year determined by mite-infestation levels through routine observations and mite monitoring carried out by beekeepers.

In contrast, some countries like South Africa [10] and Cuba [11] took a decision not to treat *Varroa* and allowed mite-resistance to appear naturally. In both countries 1,000's of colonies were lost initially, but losses declined after several years as resistance to the mite developed.

Whereas, in Brazil the evolution of mite-resistance by Africanised bees (*Apis mellifera scutellata*) was not observed as it was probably masked by the losses from untreated non-resistant European honeybee colonies. Despite this *Varroa* resistance rapidly became widespread in these countries, hence treatment-free beekeeping has been established for several decades.

More recently in mainland Europe, [12,13] the UK, [14] and USA [15] it appears an increasing number of mite-resistant populations are being managed treatment-free. Allowing honeybees to develop natural resistance will foster a long-term solution to the *Varroa* problem.

Resistance is defined as any situation in which *Varroa* populations are maintained at a suitable level for the long-term survival of the honeybee colonies [16]. Recently, three key traits (cell recapping, mite infertility and brood removal) have been associated with natural resistance in almost all resistant populations studied in different countries [17]. In the UK the government advice is to treat *Varroa* with either biotechnical methods, with registered varroacides or a combination of them [18]. Despite this, anecdotal evidence from beekeepers suggests that numbers of non-treating beekeepers are more than expected [19].

The aim of this study was therefore to conduct an online survey of UK beekeepers via their associations to assess individual treatment habits, as it was the most efficient way to reach beekeepers easily. The survey will provide crucial empirical data to support or refute common perceptions around treatment and non-treatment beekeeping practices.

Materials and methods

The survey

The survey was constructed using Google Forms as it allowed an unlimited number of responses whilst also allowing the incorporation of the University logo to add credibility to the survey. The survey consisted of a brief description outlining the study, its aims, and our definition of treatment, which was, "any form of external or additional control administered to bees by beekeepers aimed at reducing *Varroa* numbers". Then followed six questions; 1) association name, 2) number of colonies, 3) if they treat or not, 4) number of times a year they treat, 5) number of years since last treatment, 6) type of treatment (see Fig S2 for survey). Answers were either multiple-choice questions or open questions that all helped assess the beekeeper's treatment habits. The survey was kept short in duration to increase the response rates [20].

The contact details for 325 beekeeping area associations across the UK (Fig. 1a) were obtained via the four UK Beekeeper Association websites (British, Scottish, Welsh and Northern Ireland). Initial contact was made by email with 303 of them; outlining the study aims to determine general interest. The responding associations were then sent the survey online link and asked to forward it to their respective members. Beekeepers were given ten weeks from 23rd Dec 2020 to complete the survey. A small number of beekeepers did not complete every section of the survey fully, so for each analysis the precise number of responses obtained from that part are given. Amateur bee-associations were chosen as they contain around 30,000 members in the UK whereas there are around 400 bee framers in the UK.

The survey analysis

After the survey closed, all 2,897 responses from 243 associations were exported into an excel spreadsheet. The 25 responses from the Isle of Man, which remains a *Varroa*-free island were excluded along with the 16 beekeepers with zero colonies leaving 2856 for further analysis. A total of 67 beekeepers did not either belong to or failed to put down an association. As over 20 types of *Varroa* treatment were reported, we grouped them into single methods (Chemical, Biomechanical and Natural) and mixed methods.

Spatial analysis

Maps were created using ArcGIS Pro (v. 10.8.1) software, showing the location of all UK area associations and those that participated (Fig 1a). To investigate if any spatial patterns in treatment-free vs treated existed, the proportion of beekeepers falling into either category were calculated from each association that had at least five responses to avoid illegible pie diagrams on the figure (Fig.1b)

Statistical analysis

A GLM was conducted in R [21] using treatment status as the predictor, association as the fixed effect and colony number as the random effect. A binomial family was used to fit the model due to only two variants of the response variable (i.e., treating or treatment-free).

Results

The 2,856 beekeepers who responded represents almost 10% of the estimated 30,000 members belonging to the four UK beekeeping associations and were wildly distributed across the UK (Fig. 1a). An estimate of the number of colonies surveyed was calculated by multiplying the median colony group size (Table 1) by the number of beekeepers in that group, which suggests 21,200 colonies surveyed that is around 10% of the 220,000 colonies estimated by the Center for Ecology and Hydrology to be in the UK.

[insert Fig. 1 around here]

Fig. 1. A) Distribution of all 325 UK beekeeping area associations (blue dots) and the 158 area associations used in the spatial study (black dots). B) Distribution and relative proportion of Varroa-treating (purple) and treatment-free beekeepers (pale blue) at an associations level across the UK. Larger pies indicate more responses from that association. Map image is the intellectual property of Esri and is used herein under license. Copyright © 2020 Esri and its licensors. All rights reserved. Esri, USGS | Esri UK, Esri, HERE, Garmin, FAO, NOAA, USGS.

The majority (67%) of beekeepers that responded managed between 1 to 5 colonies and only 3% had over 30 colonies (Table 1). Our data indicated that across the five colony size groups the proportion of treatment-free beekeepers was greatest in the 1-5 group, although all other groups were within 5% of that group (Table 1). The GLM (Table 2) showed that treatment status was not affected by association but did indicate a significant effect of colony group size on treatment status. This due to a significant difference between the 1-5 and 6-15 groups ($X_{2\ [1,\ n=2628]}$ = 9.81, p =.0017).

Table 1. The number of colonies, shown in four groups, managed by the responding beekeepers. The percentage of each group relative to the total is also given. Also the number of treatment-free beekeepers with that group are presented along with the percentage of the that group.

Colony number groups	No. of beekeepers (% of respondents)	No. of treatment-free beekeepers (% of treatment-free beekeepers in group)
1-5	1,912 (67%)	430 (23%)

6-15	716 (25%)	121 (17%)
16-30	139 (5%)	28 (20%)
30+	89 (3%)	17 (19%)
Total	2,856	596 (21%)

Table 2. A generalized linear model (GLM) showing the association had not effect on colony number but there was a significant effect of colony number on treatment status. The range of the Z and Pr values for associations are given rather than the long list of individual values.

	Estimate	Std. error	Z value	Pr(>|z|)
Intercept	--1.828E+01	2.463E+03	-0.007	0.994
Colony number group	-1.815E-02	7.951E-03	-2.282	0.0225*
Association	-9.647E-02-3.703E+01	2.463E+03-6.972E+03	0-0.012	0.993-1

*<0.05

Treatment-free beekeeping

A total of 596 (21%) beekeepers stated they were not treating, whereas 2,260 (79%) beekeepers were treating their colonies against *Varroa*. When asked about the duration elapsed since their last treatment was applied, 72% had treated within the last year and 166 (6%) responders had not treated for 10 years or never treated (Table 3). The spatial distribution (Fig. 1b) indicates Scottish beekeepers were almost all treating and only four associations had more than 75% of treatment-free beekeepers as members, otherwise

treatment free beekeepers were spread mainly throughout England. Thus, indicating the widespread distribution of beekeepers attempting treatment free approaches particularly in England.

Table 3. Number and percentage of responses for the time since beekeepers last treated their colonies for *Varroa*. Enclosed in parentheses is the year ranges in which the last treatments were administered.

Last treatment administered	N	Percentage of total (%)
<1 year (2020-2021)	2,046	72
1-5 years (2016-2020) *	536	19
6-10 years (2016-2011)	108	4
10+ (2011 or earlier)	65	2
Never administered a treatment	101	4
Total	2,856	

* Some beekeepers in this group may treat if their colonies become overwhelmed with mites, but at the time of the survey it indicates the intention is not to treat their colonies.

Treatments used in the UK

An estimated 4,093 treatments per year were administered by 2,238 beekeepers with the aim of reducing *Varroa* numbers. The majority (70%) are treated once or twice a year (Table 4) using a single chemical method (Table 5). The most popular chemical treatment method is Oxalic acid, followed by commercially produced Thymol, and Amitraz. The current study found 78% of beekeepers use chemical treatments (Oxalic acid, Thymol etc), 3% use biomechanical methods (drone brood removal, sugar dusting etc) and less than 1% use other

methods (rhubarb leaves, etc.) (Table 5). Overall, 80% use a single method of treatment and only 20% use a combination of treatment methods (Table 5).

Table 4. Treatment application data outlining the number of responses for each application category and subsequent number of treatment applications applied to *Apis mellifera* with the aim of reducing *Varroa destructor* mite numbers.

Applications per year	N	Percentage of total (%)
0 (i.e., treatment free)	596	21
1	864	30
2	1,148	41
3+	226	8
Total	2,834*	

* a small number of beekeepers failed to report what treatment type they used.

Table 5. The number of treatment applications per year from 2,238 beekeepers across the UK grouping treatments into single compounds or methods, along with beekeepers those using a combination of methods.

Treatment Category	% of total treatments
**Active ingredients used in single applications 80%**	
**Chemical**	
Oxalic acid	31.4
Thymol	19.3
Amitraz	18.3
Formic acid	10.8
Tau-fluvalinate	3.7
Flumethrin	0.2
Others	0.2
**Biomechanical**	
Drone brood removal	5.4
Sugar/flour dusting	5.1
Shook swarm	1.6
False/Mesh floor	1.5
Bee gym	0.6
Brood breaks	0.4
Changing frames	0.2
Queen trapping	0.2
**Natural methods**	

Rhubarb leaves	0.3
Thyme oil	0.2
Crushed garlic	0.1
Homeopathic sprays	0.2
Scented leaves	0.1
Eucalyptus oil	0.1
Other essential oils	0.1
**Mixed treatments 20%**	
Chemical/ biomechanical	17.1
Biomechanical/natural	0.3
Chemical/natural	0.4
Chem/bio/natural	0.2
**Total number of treatments**	**4,093**

Discussion

Based on 2,856 beekeeper responses from 243 UK beekeeping areas, the proportion of treatment-free beekeeping ranged between 21-28% e.g., if the beekeeper can only choose between treatment or treatment-free (21%), or 28% of beekeepers if you include those that had not treated in the last year. Those not treating for over 6 years or have never treated, represented 10% of responders. Based on this survey, that would mean 3,000 of the 30,000 estimated UK beekeepers may have never or are not treating their colonies for *Varroa* for six years or more. Around 100 of these beekeepers are in a single region in North Wales [22] with many belonging to the Lleyn and Eifionydd Beekeepers' Association. Likewise, in Swindon, a small beekeeper group have kept treatment-free colonies since 1995 [23]. However, many UK beekeepers are often hesitant to reveal that they are not treating as they are in the minority and those not treating have been seen as ‘bad’ beekeepers by those treating. This may explain the 67 beekeepers that did not declare an association due to their unorthodox treatment method or lack of any mite-treatment method. In the UK there are active and growing “treatment-free” communities. For instance, the Westerham beekeepers are approaching their 5th year of becoming treatment free and starting to bring in neighboring

bee clubs. Finally, the ‘Natural Beekeeping Trust’ has links to 35 like-minded groups and a small number of their members fall into the ‘never-treated group’, but these beekeepers have not always been welcomed by those adopting treatment regimens [24]. The study indicates that despite hesitation there appears to be a growing number of beekeepers that already have or are switching from using treatments to becoming treatment-free over the past decade, although their numbers remain small relative to those treating. These treatment-free beekeepers’occur at a similar proportion (17-23%) irrespective of the number of colonies they manage.

The annual BBKA (British Beekeepers Association) overwinter survival survey found that in 2020/21, 27% of 2,950 randomly selected members from a pool of 26,407 did not treat between August and September, which is the main treatment period in the UK [6]. In 2019/20 the BBKA survey found 25% of treatment-free beekeeping [25] and supports the level and widespread distribution of treatment-free beekeeping found by this study (Fig. 1b). This is important, since the two surveys used different sampling methods. The BBKA and this study surveyed similar numbers of beekeepers, except the BBKA survey targets members randomly selected from their membership each year, whereas this study approaches the beekeepers via their associations using email so are self-selecting. Despite this the two outcomes are similar in many aspects indicating that the survey has not disproportionally been returned by treatment-free beekeepers that may be more active online. In fact, the opposite is the case since the BBKA survey reported significantly more (X_2 = 29.3, p<.001) treatment-free beekeepers than the current survey

Nonetheless, the majority (72-79%) of UK beekeepers are still treating their colonies to control *Varroa* numbers. We found beekeepers in the UK are predominantly using a single chemical treatment, with the order of popularity been Oxalic acid, Thymol, Amitraz and

Formic acid. These treatments were used once or twice yearly. Only 20% of beekeepers in this study are adopting a combination of methods approach (Table 5) although treatment decisions appear to be based on seeing mites on bees or on the floor which is surprising given the high amounts of endorsement that IPM receives [9, 26, 27]. The use of one method may be more appealing to beekeepers because it is less time consuming than attempting a combination of methods over an extended period such as in an IPM strategy.

The popular chemical *Varroa* treatments like Formic and Oxalic acids reported in this study and other UK surveys [6, 25] are also the preferred methods used in Europe [28] and the USA [29]. These compounds have a high efficacy but without the stigma of synthetic compounds or mite resistance, which could explain why many beekeepers are choosing to adopt these methods [30]. However, the impact of these "natural" treatments should not be ignored. Whilst Thymol is thought to be unproblematic at temperatures between 5°C-9°C, high mortality levels have been observed when temperatures exceed 27°C [28, 31, 32].

The success of any treatment method or treatment-free beekeeping is determined by their overwinter losses, over the last 14 years in the UK over-wintering annual colony losses (Fig. S1) have ranged from over 30% to 8% with an average of 18%: which falls to 14% when the three spikes related to unusually cold and wet winters are removed (data derived from BBKA annual survey data collected by D. Aston based on 2,500-3,500 beekeepers each year [e.g. 6, 25]). Principal causes of colony losses reported by beekeepers in 2021 were Queen-related problems (24%), Isolation starvation (21%), Weather-related 17%, and *Varroa* were just 4% [6] reflecting the situation that the mite is no longer considered a major problem by beekeepers currently in the UK. However, *Varroa* could be contributing to some of the other colony losses indirectly. In 2019-2020 the BBKA annual survey reported both losses in the region as well as the percentage of colonies not treated in each region [25]. We found no

significant correlation in this data between not treating and colony loss rates across the seven UK regions. ($r_{(5)}$=.44 p=.32).

A survey conducted by the Oxfordshire Natural Beekeeping Association [33] (ONBA), found their colony survival rates were on par with those engaging in treatment methods. For example, during the last two years (2018-2019 and 2019-2020), losses were 8% (ONBA) vs 9% (BBKA) and 18% (ONBA) vs 17% (BBKA) respectively. Furthermore, the treatment-free group in Northwest Wales, reported an average 13% winter losses from 2012-2015 as compared to 19% for treated colonies [22].

One of the dangers of switching over to an intervention free-treatment regime involves a period of high winter losses whilst the bees develop resistance [34]. This is due to re-invasion from collapsing colonies to surviving surrounding colonies [24] or by visits from infested non-natal bees [35]. Several strategies have been adopted at both local and national level to encourage mite-resistant honeybees. Catching free-living swarms from locations where they appear to have persisted for many years has been a successful strategy in the UK [22] and Hawaii [36]. Others have reduced the frequency of mite-treatments by selectively treating colonies with high mite levels, or to use less efficient biomechanical methods only when required.

Already many countries (South Africa, Brazil, Mexico, Cuba etc), along with beekeepers in the UK and elsewhere have been able to stop treating as their honeybees have learnt to detect mite infested cells and remove the pupa to prevent mite-reproduction, which leads to decreased mite fertility and population size. However, the majority (72-79%) of UK bees are still treating thus it will be many years before most beekeepers in the UK and elsewhere can

stop treating for *Varroa*. In the USA a recent survey found only around 63 (3%) of 2275 respondents stated no advantages to *Varroa* management and 92% of this group did not treat for *Varroa* [37], a very different situation than found in currently in the UK. Although, most beekeepers in the Northern hemisphere have long wished for a silver bullet for the *Varroa* problem, however, it turns out that the bullet is their own bees, they just need to give their bees time to develop mite resistant as has been done so successfully elsewhere in the world.

Acknowledgments

We thank all the UK beekeepers that submitted a survey, help from their associations and G. Webb of University of Salford for proofreading the original submission. The original data is available on dryad.

Author Contributions

Conceptualization: Stephen J Martin, Alexandra Valentine

Data curation: Alexandra Valentine

Formal analysis: Alexandra Valentine

Funding acquisition: Stephen J Martin

Investigation: Alexandra Valentine

Methodology: Alexandra Valentine, Stephen J Martin

Project administration: Stephen J Martin, Alexandra Valentine

Resources: Stephen J Martin

Supervision: Stephen J Martin

Validation: Alexandra Valentine

Visualization: Alexandra Valentine

Writing – original draft: Alexandra Valentine

Writing – review & editing: Stephen J Martin, Alexandra Valentine

References:

1. Genersch E. Honeybee pathology: current threats to honeybees and beekeeping. Appl Microbiol Biotechnol. 2010;87: 87-97.

2. Gallai N, Salles J-M, Vaissière BE. Economic valuation of the vulnerability of world agriculture confronted with pollinator decline. Ecol Econ. 2009; 68 (3) 810–821.

3. Genersch E, Von Der Ohe W Kaatz, H Schroeder, A Otten, C, Büchler, R. et al. The German bee monitoring project: a long-term study to understand periodically high winter losses of honeybee colonies. Apidologie. 2010;41: 332-352

4. Rosenkranz P, Aumeier P, Ziegelmann, B. Biology and control of *Varroa destructer*. J Invertebr Pathol. 2010;103: S96–S119.

5. Thompson CE, Biesmeijerb JC, Allnutt TR, Pietravalle S, Budge GE. Parasite Pressures on Feral Honeybees (*Apis mellifera sp.*). PLoS One. 2014;9: (8), e105164.

6. Aston D. Colony Survival Survey 2020-2021. British Beekeepers Association BKKA News. 2021; 228: 222.

7. Bruckner S, Steinhauer N, Engelsma J, Fauvel AM, Kulhanek K, Malcom E. et al. for the Bee Informed Partnership. 2019-2020 Honey Bee Colony Losses in the United States: Preliminary Results. 2020; Available from: https://beeinformed.org/wp-content/uploads/2020/06/BIP_2019_2020_Losses_Abstract.pdf

8. Evans JD, Saegerman C, Mullin C, Haubruge E, Nguyen BK. Et al. Colony collapse disorder: a descriptive study. PloS One. 2009;4 : (8), e6481.

9. Roth MA, Wilson JM, Tignor KR, Gross AD. Biology and management of *Varroa destructor* (Mesostigmata: Varroidae) in *Apis mellifera* (Hymenoptera: Apidae) colonies. J Integr Pest Manag. 2020;11: (1), 1.

10. Allsopp MH. Analysis of *Varroa destructor* infestation of southern African honeybee populations. 2006: MSc dissertation. University of Pretoria, Pretoria, South Africa.

11. Rodriguez A, Yadro AC, Perez A, Invernizzi C, Tomasco I. Characterization of *Varroa destructor* mites in Cuba using mitochondrial and nuclear markers. J Apic Sci. 2020; 64; 335-343.

12. Oddie M, Büchler R, Dahle B, Kovacic M, Le Conte Y, Locke B. et al. Rapid parallel evolution overcomes global honeybee parasite. Sci Rep. 2018;8 :(1), 1-9.

13. Mondet F, Beaurepaire A, McAfee A, Locke B, Alaux C, Blanchard S. et al. Honey bee survival mechanisms against the parasite *Varroa destructor*: a systematic review of phenotypic and genomic research efforts. Int J Parasitol. 2020;50: 433–47.

14. Hawkins GP, Martin SJ. Elevated recapping behaviour and reduced *Varroa destructor* reproduction in mite resistant *Apis mellifera* honeybees from the UK. Apidologie 2021;52: 647–657.

15. Kulhanek K, Steinhauer N, Wilkes J, Wilson M, Spivak M. et al. (2021) Survey-derived best management practices for backyard beekeepers improve colony health and reduce mortality. PLoS One. 2021;16 :(1), e0245490.

16. Mondragón L, Spivak M, Vandame R. A multifactorial study of the resistance of honeybees *Apis mellifera* to the mite *Varroa destructor* over one year in Mexico. Apidologie 2005;36 : (3), 345-358.

17. Grindrod I, Martin SJ. Parallel Evolution of *Varroa* Resistance in Honeybees; a common mechanism across continents? Proc R Soc B. 2021; 288: 20211375.

18. Brown M, Learner J, Royle G, Wilford J. Managing *Varroa*. The National Bee Unit Animal, Animal and Plant Health Agency, UK. 2020: Published by UK Dept of Food and Argicul.

19. Heaf D. Treatment Free Beekeeping. 2021: Northern Bee Books, UK pp 122. ISBN 978-1913811006.

20. Rolstad S, Adler J, Rydén A. Response burden and questionnaire length: is shorter better? A review and meta-analysis. Value in Health. 2011;14 :(8), 1101-1108.

21. R Core Team. R: A language and environment for statistical computing. 2021; R Foundation for Statistical Computing, Vienna, Austria. URL https://www.R-project.org/.

22.Hudson C, Hudson S. Treatment-free beekeeping. British Beekeepers Association News 2020; 277: 229–232.

23. Hoskins R. Swindon Honeybee Conservation Group. 2014; Available from: http://www.swindonhoneybeeconservation.org.uk

24. Andrews E. To save the bees or not to save the bees: honeybee health in the Anthropocene. Agric. Hum. Values. 2019; 36: (4), 891-902.

25. Aston D. Winter Survival Survey Results 2019-2020. British Beekeepers Association News. 2020; 227: 244-245.

26. Jack C, Ellis JD. Integrated pest management control of *Varroa destructor* (Acari: Varroidae), the most damaging pest of (*Apis mellifera* L. [Hymenoptera: Apidae]) colonies. J Insect Sci. 2021;21: 1-32.

27. Vilarem C, Piou V, Vogelweith F, Vétillard A. *Varroa destructor* from the Laboratory to the Field: Control, Biocontrol and IPM Perspectives—A Review. Insects. 2021;12: 800.

28. Tihelka E. Effects of synthetic and organic acaricides on honeybee health: A review. Slov Vet Res. 2018;55: (3), 22.

29. Millán- Leiva A, Marín Ó, Christmon, K, vanEngelsdorp D, González- Cabrera J. Mutations associated with pyrethroid resistance in *Varroa* mite, a parasite of honeybees, are widespread across the United States. Pest Manag. Sci. 2021;77: (7), 3241–3249.

30. Underwood RM, Currie RW. The effects of temperature and dose of formic acid on treatment efficacy against *Varroa destructor* (Acari: Varroidae), a parasite of *Apis mellifera* (Hymenoptera: Apidae). Exp Appl Acarol. 2003;29: (3), 303-313.

31. Gal H, Slabezki Y, Lensky Y. A preliminary report on the effect of origanum oil and thymol applications in honeybee (*Apis mellifera L.*) colonies in a subtropical climate on population levels of *Varroa jacobsoni*. Bee Sci. 1992;2 :(4) 175-180.

32. Ellis MD, Baxendale FP. Toxicity of seven monoterpenoids to tracheal mites (Acari: Tarsonemidae) and their honeybee (Hymenoptera: Apidae) hosts when applied as fumigants. J Econ Entomol. 1997;90 :(5) 1087-1091.

33. Oxfordshire Natural Beekeeping Group. Winter survival surveys show 'treatment-free' works [online] 2020: Available from: https://oxnatbees.wordpress.com/2020/08/05/winter-survival-surveys-show-treatment-free-works/.

34. Büchler R, Berg S, Le Conte Y. Breeding for resistance to *Varroa destructor* in Europe. Apidologie. 2010;41: (3) 393-408.

35. Kulhanek K, Garavito A, vanEngelsdorp D. Accelerated *Varroa destructor* population growth in honeybee (*Apis mellifera*) colonies is associated with visitation from non-natal bees. Sci Rep. 2021; 11**:** 7092.

36. Martin SJ. Naturally mite-resistant colonies evolve on Hawaii. Am. Bee J. 2020; 16: 649-651.

37. Thoms CA, Nelson KC, Kubas A, Steinhauer N, Wilson ME, vanEngelsdorp D. Beekeeper stewardship, colony loss, and *Varroa destructor* management. Ambio. 2019; 48: 1209-1218.

Figure

Click here to access/download;Figure;Fig 1 updated.pdf

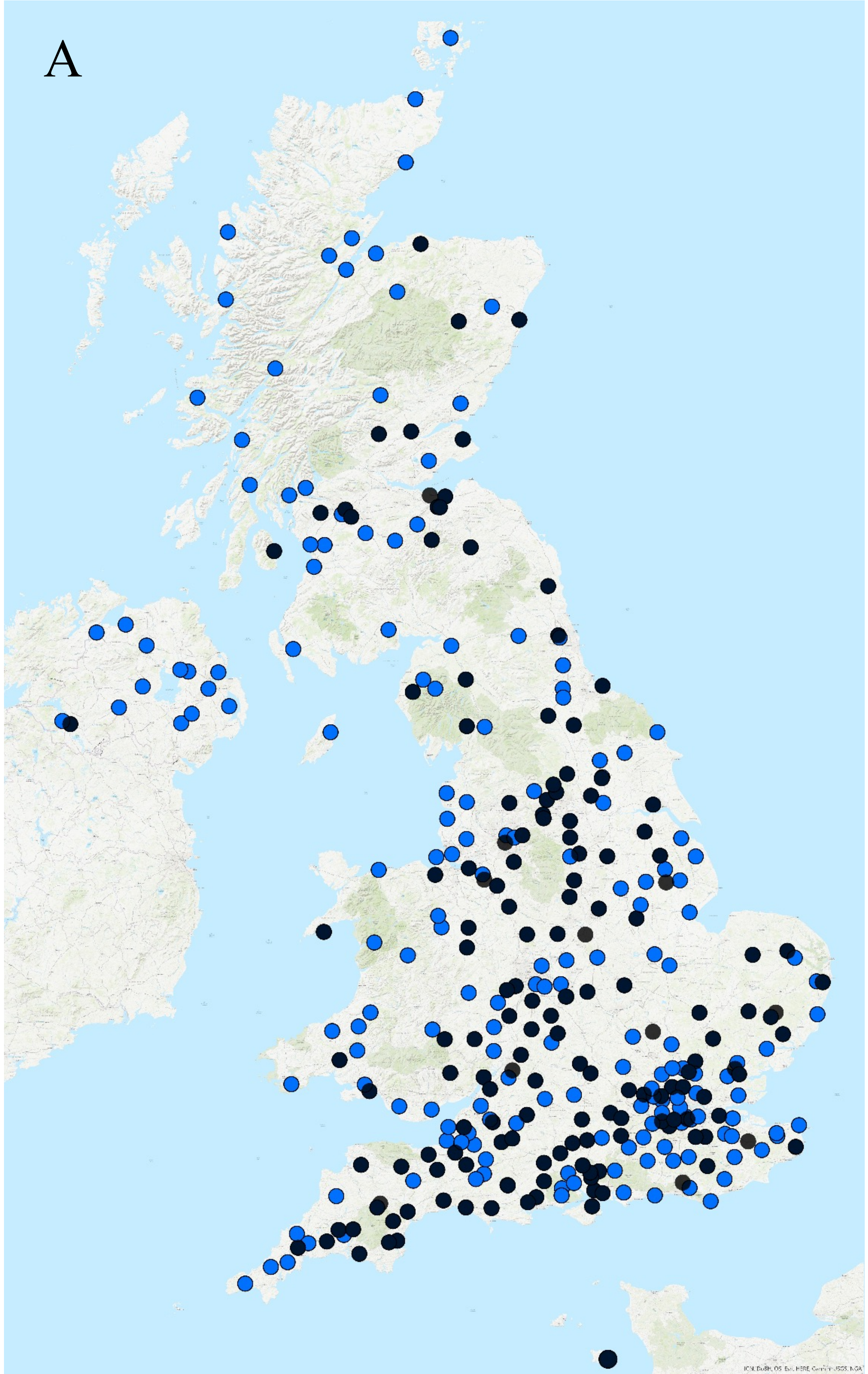

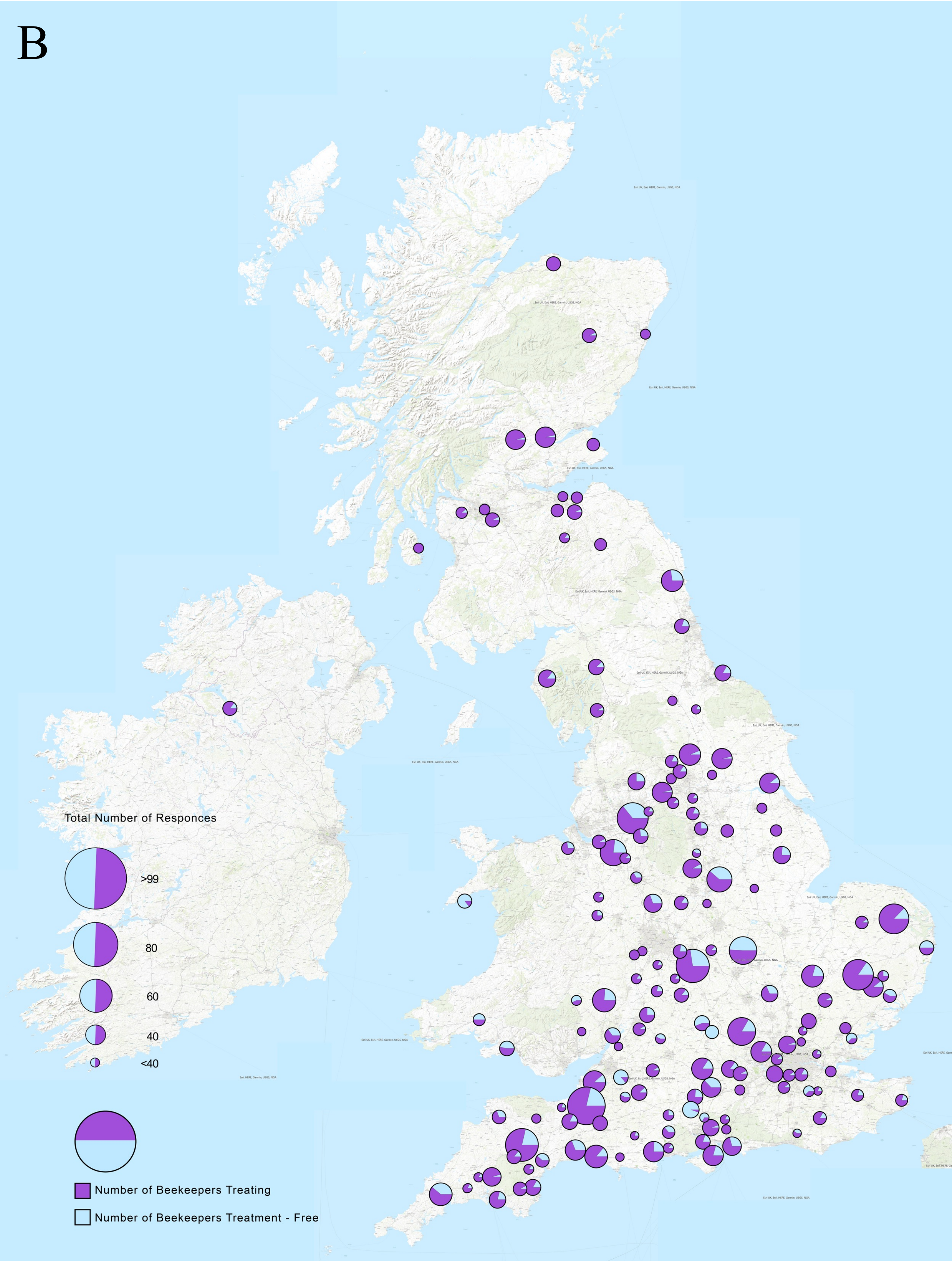

Click here to access/download
Supporting Information
Supplementary Data.docx

A survey of UK Beekeeper's *Varroa* treatment habits

Alexandra Valentine[1,2] and Stephen John Martin[1]*

1 School of Science, Engineering and Environment, The University of Salford, M5 4WT, Manchester, UK

2 Current address *School of Natural Sciences, National University of Ireland Galway, Ireland*

a.valentine2@nuigalway.ie

*** Correspondence to; s.j.martin@salford.ac.uk** orcid.org/0000-0001-9418-053X

Abstract

The global spread of the parasitic mite *Varroa destructor* instigated a substantial decline in both managed and feral honeybee (*Apis mellifera)* colonies mainly across the Northern hemisphere. In response, many beekeepers began to treat their colonies with chemical acaricides to control mite populations in managed colonies. However, some countries or beekeepers allowed their bees to develop mite-resistance by adopting a “treatment-free” approach, rather than using selective breeding programs. Yet, the distribution and proportion of beekeepers either treating or not within the United Kingdom (UK) is unknown, as it is in any Northern hemisphere country. Therefore, the aim of this study was to conduct a beekeeper survey to determine the current treatment strategies within the UK. We gathered 2,872 beekeeper responses from an estimated 30,000 UK beekeepers belonging to 242 bee-associations in the winter of 2020/21. The survey indicated that the majority (72-79%) of UK beekeepers are still treating their bees for *Varroa*, typically twice-yearly using chemical-based methods. Despite this, ~~ese~~ an estimated 3,000 UK beekeepers are treatment free for over 6 years or have never treated. This is reflected by our finding that only 78 associations out of 242 consist of responders who entirely treated. ~~Furthermore, a marked significant increase in treatment-free beekeepers occurred as the number of colonies they managed increased.~~ Although overall treatment status was not affected by association currently, we predict that in ~~. In~~ time the shift towards intervention-free approaches could lead to increasing numbers of *Varroa*-resistant ~~feral and~~ managed UK honeybee colonies.

Formatted: Font: Bold

Introduction

Honeybees are key pollinators of crops and wildflowers [1]. They contribute globally more than €53 billion annually to the economy [2]. Still, honeybees are presently facing multiple stressors such as habitat destruction, pests, and pathogens. Across the Northern hemisphere the spread of the ecto-parasitic mite *Varroa destructor* (*Varroa*) and the key viral pathogen it transmits (Deformed Wing Virus) is the number one threat faced by beekeepers over past decades, causing an increase in over-wintering losses [3, 4]. In the UK, this was evidenced in the very high losses of managed colonies and the near eradication of feral colonies within a few years of *Varroa* becoming established [5]. Although,~~The initial high~~ over-wintering colony losses have been declining in the UK during the past decade's (Fig. S1) since *Varroa*-treatments became ~~widspread~~widespread- [6]. Although colony losses remain~~ed~~ consistently high in the USA [7], which is unexpected due to the high~~almost universal~~ levels of treatment carried out by beekeepers to control *Varroa* populations.

Formatted: Font: Italic

Typically, a variety of highly effective acaricides are primarily used to control *Varroa* populations, but with the development of acaricide resistance [8] alternative commercially based treatments established~~ based~~ around T~~t~~hymol and organic acids (O~~o~~xalic and F~~f~~ormic) haves~~ ~~ become increasingly popular~~increasingly been used~~. More time consuming and often less effective biomechanical treatment methods can also be used, such as powdered sugar and drone trapping. The application of Integrated Pest Management (IPM) techniques is suggested to be the most~~re~~ effective way [9], to treat mites since it~~which~~ involves a variety of methods applied in rotation throughout the year determined by mite-infestation levels through routine observations and mite monitoring carried out by beekeepers. ~~This strategy could be beneficial for the long-term resistance of honeybees because the workers may be able to recover from low mite infestations independently.~~

In contrast, some countries like South Africa [10] and Cuba [11] took a decision not to treat *Varroa* and allowed mite-resistance to appear naturally. ~~Initially,~~ I~~i~~n both countries 1,000's of colonies were lost~~-~~ initially~~to begin with~~, but losses declined after several years as resistance to the mite developed. Whereas, in Brazil the evolution of mite-resistance by Africanised bees *(Apis mellifera scutellata)* was not observed~~vered~~ as it was probably masked by the losses from untreated non-resistant European honeybee colonies. Despite this *Varroa* resistance rapidly became widespread in these countries, hence treatment-free beekeeping has been established for several decades.

More recently in mainland Europe, [12,13] the UK, [14] and the USA [15] it appears an increasing number of mite-resistant populations are being managed treatment-free. Allowing honeybees to develop natural resistance will foster a long-term solution to the *Varroa* problem.

Resistance is defined as any situation in which *Varroa* populations are maintained at a suitable level for the long-term survival of the honeybee colonies [16]. Recently, three key traits (cell recapping, mite infertility and brood removal) have been associated with natural resistance in almost all resistant populations studied in different countries [17]. In the UK the g~~G~~overnment advice is to treat *Varroa* with either b~~B~~iotechnical methods, ~~or~~ with registered varroacides or a combination of them [18]. Despite this, anecdotal evidence from beekeepers suggests that numbers of non-treating beekeepers are more than expected [19].

The aim of this study was therefore to conduct an online survey of UK beekeepers via their associations to assess individual treatment habits, as it was the most efficient way to reach

Formatted: Font: Italic

Formatted: Font: Italic

Formatted: Font: Italic

beekeepers easily. The survey will provide crucial empirical data to support or refute common perceptions around treatment and non-treatment beekeeping practices.

Materials and methods

The survey

The survey was constructed using Google Forms as it allowed an unlimited number of responses whilst also allowing the incorporation of the University logo to add credibility to the survey. The survey consisted of a brief description outlining the study, its aims, and our definition of treatment, which was, "any form of external or additional control administered to bees by beekeepers aimed at reducing *Varroa* numbers". Then followed six questions; 1) association name, 2) number of colonies, 3) if they treat or not, 4) number of times a year they treat, 5) number of years since last treatment, 6) type of treatment (see Fig S21 for survey). Answers were either multiple-choice questions or open questions that all helped assess the beekeeper's treatment habits. The survey was kept short in duration to increase the response rates [20].

The contact details for 325 beekeeping area associations across the UK (Fig. 1) were obtained via the four UK Beekeepers Association websites (British, Scottish, Welsh and Northern Ireland). Initial contact was made by email with 303 of them; outlining the study aims to determine general interest. The responding associations were then sent the survey online link and asked to forward it to their respective members. Beekeepers were given ten weeks from 23rd Dec 2020 to complete the survey. A small number of beekeepers did not complete every section of the survey fully, so for each analysis the precise number of responses obtained from of that part are given. Amateur bee-associations were chosen as their contain around 30,000 members in the UK whereas there are around 400 bee framers in the UK.

The survey analysis

After the survey closed, all 2,897 responses from 243 associations were exported into an excel spreadsheet. The 25 responses from the Isle of Man, which remains a *Varroa*-free island were excluded along with the 16 beekeepers with zero colonies leaving 2856~~72~~ for further analysis~~from the 2,897 responses~~. A total of 67 beekeepers did not either belong to or failed to put down an association. As over 20 types of *Varroa* treatment were reported, we grouped them into single methods (Chemical, Biomechanical and Natural) and mixed methods~~ (Table S1)~~.

Spatial analysis

Maps were created using ArcGIS Pro (v. 10.8.1) software, showing the location of all UK area associations and those that participated (Fig 1). To investigate if any spatial patterns in treatment-~~-~~free vs treated existed, the proportion of beekeepers falling into either category were calculated from each association that had at least five responses to avoid illegible pie diagrams on the figure.

Statistical analysis

A GLM was conducted in R [21] using treatment status as the predictor, a~~A~~ssociation as the fixed effect and colony number as the random effect. A binomial family was used to fit the model due to only two variants of the response variable (i.e., treating or treatment-free).

Results

The 2,856~~72~~ beekeepers who responded represents almost 10% of the estimated 30,000 members belonging to the four UK beekeeping associations and were wildly distributed across the UK (Fig. 1A.). An estimate of the number of colonies surveyed was calculated by multiplying the median colony group size (Table 1) by the number of beekeepers in that

group, which produces 21,200 colonies surveyed that is around 10% of the 220,000 colonies estimated by the Center for Ecology and Hydrology to be in the UK.

[Fig. 1]

The majority (678%) of beekeepers that responded managed between 1 to 5 colonies and only 3% had over 30 colonies (Table 1). OInterestingly our data indicated that across the five colony size groups the proportion of treatment-free beekeepers was greatest in the 1-5 group, although all other groups were within 5% of the highest group similar ranging from 17% to 2392% (Table 1).of the 1-5 colony group consisted of beekeepers that treated their colonies. Whereas the most treatment-free beekeepers fell into the 16-30 colony group. The GLM (Table 2) showed that treatment status was not affected by association but did indicate a significant effect of colony group size on treatment status. This due to a significant difference between the 1-5 and 6-15 groups ($X_{2\ [1,\ n=2628]}$ = 9.81, p =.0017).This explained why there was a significant association with colony number and treatment statusincrease in treatment-free beekeepers as the numbers of colonies they managed increased, although treatment status was not affected by association (Table 2).

Formatted: Subscript

Formatted: Highlight

Table 1. The number of colonies, shown in four groups, managed byfrom the responding beekeepers but excluding theand doesn't include the 16 participants with zero colonies. The percentage of each group relative to the total is also given. Also the number of treatment-free beekeepers with that group are presented along with the percentage of the that group.. The results of the GLM are also givening indicating the percentage of treatment-free beekeepers in each group of colony numbers.

Colony number groups	No. of beekeepers (% of respondents)	No. of tTreatment-free beekeeperscolonies (% of treatment-free beekeepers in groupf group size)
1-5	1,912 (67%)	430 (238%)
6-15	716 (25%)	121 (1743%)
16-30	139 (5%)	28 (2073%)
30+	89 (3%)	17 (1958%)
Total	2,856	596 (21%)

Table 2. A generalized linear model (GLM) showing the association had not affect on colony number but there was a significant effect of colony number on treatment status. The range of the Z and Pr values are given rather than the long list of individual values.

	Estimate	Std. error	Z value	Pr(>|z|)
Intercept	--1.828E+01	2.463E+03	-0.007	0.994
Colony number group	-1.815E-02	7.951E-03	-2.282	0.0225*
Association	-9.647E-02-3.703E+01	2.463E+03-6.972E+03	0-0.012	0.993-1

*<0.05

~~Table 2. Final generalized linear model (GLM) results investigating whether colony number and association were affecting treatment status. The range of the Z and Pr values are given rather than the long list of individual values.~~

	~~Estimate~~	~~Std. error~~	~~Z value~~	~~Pr(>|z|)~~
~~Intercept~~	~~-2.57E+01~~	~~3.31E+03~~	~~-0.008~~	~~0.994~~
~~Colony number~~	~~-2.30E+00~~	~~5.84E+03~~	~~20.723~~	~~<2e-16***~~
~~Association~~	~~-2.30E+00- 4.45E+01~~	~~3.31E+03- 1.13E+04~~	~~0-0.01~~	~~0.992-1~~

~~*** <.0001~~

Treatment-free beekeeping

A total of 596~~02~~ (21%) beekeepers stated they were not treating, whereas 2,260 (79%) beekeepers were treating their colonies against *Varroa*. When asked about the duration elapsed since their ~~time since the~~ last treatment was applied, 72% had treated within the last year and 166 (6%) responders had not treated for 10 years or never treated (Table 3~~23~~). The spatial distribution (Fig. 1B) indicates Scottish beekeepers were almost all treating and only four associations had more than 75% of treatment-free beekeepers as members, otherwise treatment free beekeepers were spread mainly throughout England. Thus, indicating the widespread distribution of beekeepers attempting treatment free approaches particularly in England.

Table 323. Number and percentage of responses for the time since beekeepers last treated their colonies for *Varroa*. Enclosed in parentheses is the year ranges in which the last treatments were administered.

Last treatment administered	N	Percentage of total (%)
<1 year (2020-2021)	2,046	72
1-5 years (2016-2020) *	536	19
6-10 years (2016-2011)	108	4
10+ (2011 or earlier)	65	2
Never administered a treatment	101	4
Total	2,856	

* Some beekeepers in this group may treat if their colonies become overwhelmed with mites, but at the time of the survey it indicates the intention is not to treat their colonies.

Treatments used in the UK

An estimated 4,093 treatments per year were administered by 2,238 beekeepers with the aim of reducing *Varroa* numbers. The majority (70%) are treated once or twice a year (Table 434) using a single chemical method (Table 545). The most popular chemical treatment method is Ooxalic acid, followed by commercially produced Tthymol, and Amitraz. The current study found 78% of beekeepers use chemical treatments (Oxalic acid, Thymol etc), 3% use biomechanical methods (dDrone brood removal, sugar dusting etc) and less than 1% use other methods (rRhubarb leaves, etcetc.) (Table 545). Overall, 80% use a single method of treatment and only 20% use a combination of treatment methods (Table 545).

Table 434. Treatment application data outlining the number of responses for each application category and subsequent number of treatment applications applied to *Apis mellifera* with the aim of reducing *Varroa destructor* mite numbers.

Applications per year	N	Percentage of total (%)
0 (i.e., treatment free)	596	21
1	864	30
2	1,148	41
3+	226	8
Total	2,834*	

* a small number of beekeepers failed to report what treatment type they used.

Table 545. The number of treatment applications per year from 2,238 beekeepers across the UK grouping treatments into single compounds or methods, along with beekeepers those using a combination of methods.

Treatment Category	% of total treatments
**Active ingredients used in sSingle aApplications 80%**	
**Chemical**	
Oxalic acid	31.4
Thymol	19.3
Amitraz	18.3
Formic acid	10.8
Tau-fluvalinate	3.7
Flumethrin	0.2
Others	0.2
**Biomechanical**	
Drone brood removal	5.4
Sugar/flour dusting	5.1
Shook swarm	1.6
False/Mesh floor	1.5
Bee gym	0.6
Brood breaks	0.4
Changing frames	0.2
Queen trapping	0.2
**Natural mMethods**	
Rhubarb leaves	0.3
Thyme oil	0.2
Crushed garlic	0.1
Homeopathic sprays	0.2
Scented leaves	0.1
Eucalyptus oil	0.1

Other essential oils	0.1
**Mixed treatments~~IXED TREATMENTS~~ 20%**	
Chemical/ biomechanical	17.1
Biomechanical/natural	0.3
Chemical/natural	0.4
Chem/bio/natural	0.2
**Total number of treatments**	**4,093**

Discussion

Based on 2,856~~72~~ beekeeper responses from 243~~2~~ UK beekeeping areas, the proportion of treatment-free beekeeping ~~is~~ ranged between 21-28% e.g., if the beekeeper can only choose between treatment or treatment-free (21%), or 28% of beekeepers that had not treated in the last year. Those not treating for over 6 years or have never treated, represented 10% of responders. Based on this survey, that would mean 3,000 of the 30,000 estimated UK beekeepers may have never or are not treating their colonies for *Varroa* for six years or more. Around 100 of these beekeepers are in a single region in North Wales [22] with many belonging to the Lleyn and Eifionydd Beekeepers' Association. Likewise, in Swindon, a small beekeeper group have kept treatment-free colonies since 1995 [23]. However, many UK beekeepers are often hesitant to reveal that they are not treating as they are in the minority and those not treating have been seen as ‘bad’ beekeepers by those treating. This may explain the 67 beekeepers that did not declare an association due to their unorthodox treatment method or lack of any mite-treatment method. In the UK there are active and growing “treatment-free” communities. For instance, the Westerham beekeepers are approaching their 5th year of becoming treatment free and starting to bring in neighboring bee clubs. Finally, the ‘Natural Beekeeping Trust’ has links to 35 like-minded groups and a small number of their members fall into the ‘never-treated group’, but these beekeepers have not always been welcomed by those adopting treatment regimens [24]. The study indicates that

despite hesitation there appears to be a growing number of beekeepers that already have or are switching from using treatments to becoming treatment-free over the past decade, although their numbers remain small relative to those treating. T~~Interestingly, t~~hese treatment-free ~~beekeepers~~beekeepers' occur at a similar proportion (17-23%) irrespective of the number of colonies they manage.~~r larger numbers of colonies than treating beekeepers. This may be since it provides a bigger pool to select from.~~

The annual BBKA (British Beekeepers Association) overwinter survival survey found that in 2020/21, 27% of 2,950 randomly selected members from a pool of 26,407 did not treat between August and September, which is the main treatment period in the UK [6]. In 2019/20 the BBKA winter loss survey found 25% over the same period [25] and supports the level and widespread distribution of treatment-free beekeeping found by this study (Fig. 1B). This is important, since the two surveys use different sampling methods. The BBKA and this study surveyed similar numbers of beekeepers, except the BBKA survey targets members randomly selected from their membership each year, whereas this study approaches the beekeepers via their associations using email so are self-selecting. Despite this the two outcomes are similar in many aspects indicating that it~~and~~ has not disproportionally been returned by treatment-free beekeepers that may be more active online. In fact, the opposite is the case since the BBKA survey reported significantly more (X_2 = 29.3, p<.001) treatment-free beekeepers than the current survey.

Nonetheless, the majority (72-79%) of UK beekeepers are still treating their colonies to control *Varroa* numbers. We found beekeepers in the UK are predominantly using a single chemical treatment, with the order of popularity been O~~o~~xalic acid, T~~t~~hymol, Amitraz and Formic acid. These treatments were used once or twice yearly. Only 20% of beekeepers in this study are adopting a combination of methods approach (Table 5~~4~~5) although treatment

decisions appear to be based on seeing mites on bees or on the floor whichThis is surprising given the high amounts of endorsement that IPM receives [9, 26, 27]. Despite this, only 20% of beekeepers in this study are adopting a combination of methods approach (Table 5). The use of one method may be more appealing to beekeepers because it is less time consuming than attempting a combination of methods over an extended period such as in an IPM strategy.

The popular chemical *Varroa* treatments like Formic and Oxalic acids reported in this study and other UK surveys [6, 25] are also the preferred methods used in Europe [28] and the USA [29]. These compounds have a high efficacy but without the stigma of synthetic compounds or mite resistance, which could explain why many beekeepers are choosing to adopt these methods [30]. However, the impact of these “natural” treatments should not be ignored. Whilst Tthymol is thought to be unproblematic at temperatures between 5°C-9°C, high mortality levels have been observed when temperatures exceed 27°C [28, 31, 32].

The success of any treatment method or treatment-free beekeeping is determined by their overwinter losses, over the last 14 years in the UK over-wintering annual colony losses (Fig. S2) have ranged from over 30% to 8% with an average of 18%: which falls to 14% when the three spikes related to unusually cold and wet winters are removed (data derived from BBKA annual survey data collected by D. Aston based on 2,500-3,500 beekeepers each year [e.g. 6, 25]). Principal causes of colony losses reported by beekeepers in 2021 were Queen-related problems (24%), Isolation starvation (21%), Wweather-related 17%, andLosses due to *Varroa* were just 4% last year [6]) reflecting the situation that the mite is no longer considered a major problem by beekeepers currently in the UK. However, indirectly *Varroa* could be contributing to some of the other colony losses indirectly. In 2019-2020 the BBKA annual survey reported both losses in the region as well as the percentage of colonies not

Formatted: Font: Italic

treated in each region [25]. We found no significant correlation in this data between not treating and colony loss rates across the seven UK regions. ($r_{(5)}$=.44 p=.32).

Formatted: Subscript

high level of treatment and high survival rates of treatment free beekeeping in the UK. A survey conducted by the OxfordshireOxford-shire Natural Beekeeping Association [33] (ONBA), found their colony survival rates were on par with those engaging in treatment methods. For example, during the last two years (2018-2019 and 2019-2020), losses were 8% (ONBA) vs 9% (BBKA) and 18% (ONBA) vs 17% (BBKA) respectively. Furthermore, the treatment-free group in Northwest Wales, reported an average 13% winter losses from 2012-2015 as compared to 19% for treated colonies [22].

One of the dangers of switching over to an intervention free- treatment regime involves a period of high winter losses whilst the bees develop resistance [34]. This is due to raid re-invasion from collapsing colonies to surviving surrounding colonies [24] or by visits by from infested non-natalivetal bees [35]. Several strategies have been adopted at both local and national level to encourage mite-resistant honeybees. Catching free-livingeral swarms from locations where they appear to have persisted for many years has been a successful strategy in the UK [22] and the HawaiiUSA [36]. Others have reduced the frequency of mite-treatments by selectively treating colonies with high mite levels, or to use less efficient biomechanical methods only when required.

Commented [A1]: Feral implies they were once managed bees, free-living applies to either managed or wild origin

Already many countries (South Africa, Brazil, Mexico, Cuba ectetc), along with beekeepers in the UK and elsewhere have been able to stop treating as their honeybees have learnt to detect mite infested cells and remove the pupa to prevent mite-reproduction, which leads to decreased mite fertility and population size. However, the majority (72-79%) of UK bees are

still treating thus it will be many years before most beekeepers in the UK and elsewhere can stop treating for *Varroa*. For example, Iin the USA a recent survey found only around 639 (3%) of 2275 respondents stated no advantages to Varroa management and 92% of this group did did not treat for Varroa [37], a very different situation than found in currently in the UK. AlthoughHowever, mostallThe beekeepers in the Northern hemisphere have long wished for a sillliver bullet for the *Varroa* problem, however, whilst whiit ch turns out that the bullet is their own bees, and they just need to give their beesm time to develop mite resistant as has been done so successfully elsewhere in the world and on a very large scale.

Acknowledgments

We thank all the UK beekeepers that submitted a survey, help from their associations and G. Webb of University of Salford for proofreading the original submission. The original data will beis available on Dryad. by request to the corresponding author due to the potentially sensitive information.

Author Contributions

Conceptualization: Stephen J Martin, Alexandra Valentine

Data curation: Alexandra Valentine

Formal analysis: Alexandra Valentine

Funding acquisition: Stephen J Martin

Investigation: Alexandra Valentine

Methodology: Alexandra Valentine, Stephen J Martin

Project administration: Stephen J Martin, Alexandra Valentine

Resources: Stephen J Martin

Supervision: Stephen J Martin

Validation: Alexandra Valentine

Visualization: Alexandra Valentine

Writing – original draft: Alexandra Valentine

Writing – review & editing: Stephen J Martin, Alexandra Valentine

References:

1. Genersch E. Honeybee pathology: current threats to honeybees and beekeeping. Appl Microbiol Biotechnol. 2010;87: 87-97.

2. Gallai N, Salles J-M, Vaissière BE. Economic valuation of the vulnerability of world agriculture confronted with pollinator decline. Ecol Econ. 2009; 68 (3) 810–821.

3. Genersch E, Von Der Ohe W Kaatz, H Schroeder, A Otten, C, Büchler, R. et al. The German bee monitoring project: a long-term study to understand periodically high winter losses of honeybee colonies. Apidologie. 2010;41: 332-352

4. Rosenkranz P, Aumeier P, Ziegelmann, B. Biology and control of *Varroa destructer*. J Invertebr Pathol. 2010;103: S96–S119.

5. Thompson CE, Biesmeijerb JC, Allnutt TR, Pietravalle S, Budge GE. Parasite Pressures on Feral Honeybees (*Apis mellifera sp.*). PLoS One. 2014;9: (8), e105164.

6. Aston D. Colony Survival Survey 2020-2021. British Beekeepers Association BKKA News. 2021; 228: 222.

7. Bruckner S, Steinhauer N, Engelsma J, Fauvel AM, Kulhanek K, Malcom E. et al. for the Bee Informed Partnership. 2019-2020 Honey Bee Colony Losses in the United States: Preliminary Results. 2020; Available from: https://beeinformed.org/wp-content/uploads/2020/06/BIP_2019_2020_Losses_Abstract.pdf

8. Evans JD, Saegerman C, Mullin C, Haubruge E, Nguyen BK. Et al. Colony collapse disorder: a descriptive study. PloS One. 2009;4 : (8), e6481.

9. Roth MA, Wilson JM, Tignor KR, Gross AD. Biology and management of *Varroa destructor* (Mesostigmata: Varroidae) in *Apis mellifera* (Hymenoptera: Apidae) colonies. J Integr Pest Manag. 2020;11: (1), 1.

10. Allsopp MH. Analysis of ~~V~~*Varroa destructor* infestation of southern African honeybee populations. 2006: MSc dissertation. University of Pretoria, Pretoria, South Africa.

11. Rodriguez A, Yadro AC, Perez A, Invernizzi C, Tomasco I. Characterization of *Varroa destructor* mites in Cuba using mitochondrial and nuclear markers. J Apic Sci. 2020; 64; ~~☹:(2)~~ 335-343.

12. Oddie M, Büchler R, Dahle B, Kovacic M, Le Conte Y, Locke B. et al. Rapid parallel evolution overcomes global honeybee parasite. Sci Rep. 2018;8 :(1), 1-9.

13. Mondet F, Beaurepaire A, McAfee A, Locke B, Alaux C, Blanchard S. et al. Honey bee survival mechanisms against the parasite *Varroa destructor*: a systematic review of phenotypic and genomic research efforts. Int J Parasitol. 2020;50: 433–47.

14. Hawkins GP, Martin SJ. Elevated recapping behaviour and reduced *Varroa destructor* reproduction in mite resistant *Apis mellifera* honeybees from the UK. Apidologie 2021;52: 647–657.

15. Kulhanek K, Steinhauer N, Wilkes J, Wilson M, Spivak M. et al. (2021) Survey-derived best management practices for backyard beekeepers improve colony health and reduce mortality. PLoS One. 2021;16 :(1), e0245490.

16. Mondragón L, Spivak M, Vandame R. A multifactorial study of the resistance of honeybees *Apis mellifera* to the mite *Varroa destructor* over one year in Mexico. Apidologie 2005;36 : (3), 345-358.

17. Grindrod I, Martin SJ. Parallel Evolution of *Varroa* Resistance in Honeybees; a common mechanism across continents? Proc R Soc B. 2021; 288: 20211375.

Formatted: Font: Italic

18. Brown M, Learner J, Royle G, Wilford J. Managing *Varroa*. The National Bee Unit Animal, Animal and Plant Health Agency, UK. 2020: Published by UK Dept of Food and Argicul.

19. Heaf D. Treatment Free Beekeeping. 2021: Northern Bee Books, UK pp 122. ISBN 978-1913811006.

20. Rolstad S, Adler J, Rydén A. Response burden and questionnaire length: is shorter better? A review and meta-analysis. Value in Health. 2011;14 :(8), 1101-1108.

21. R Core Team. R: A language and environment for statistical computing. 2021; R Foundation for Statistical Computing, Vienna, Austria. URL https://www.R-project.org/.

22.Hudson C, Hudson S. Treatment-free beekeeping. British Beekeepers Association News 2020; 277: 229–232.

23. Hoskins R. Swindon Honeybee Conservation Group. 2014; Available from: http://www.swindonhoneybeeconservation.org.uk~~**Error! Hyperlink reference not valid.**~~

24. Andrews E. To save the bees or not to save the bees: honeybee health in the Anthropocene. Agric. Hum. Values. 2019; 36: (4), 891-902.

25. Aston D. Winter Survival Survey Results 2019-2020. British Beekeepers Association News. 2020; 227: 244-245.

26. Jack C, Ellis JD. Integrated pest management control of *Varroa destructor* (Acari: Varroidae), the most damaging pest of (*Apis mellifera* L. [Hymenoptera: Apidae]) colonies. J Insect Sci. 2021;21: 1-32.

27. Vilarem C, Piou V, Vogelweith F, Vétillard A. *Varroa destructor* from the Laboratory to the Field: Control, Biocontrol and IPM Perspectives—A Review. Insects. 2021;12: 800.

28. Tihelka E. Effects of synthetic and organic acaricides on honeybee health: A review. Slov Vet Res. 2018;55: (3), 22.

Formatted: Font: Italic

29. Millán- Leiva A, Marín Ó, Christmon, K, vanEngelsdorp D, González- Cabrera J. Mutations associated with pyrethroid resistance in *Varroa* mite, a parasite of honeybees, are widespread across the United States. Pest Manag. Sci. 2021;77: (7), 3241–3249.

30. Underwood RM, Currie RW. The effects of temperature and dose of formic acid on treatment efficacy against *Varroa destructor* (Acari: Varroidae), a parasite of *Apis mellifera* (Hymenoptera: Apidae). Exp Appl Acarol. 2003;29: (3), 303-313.

31. Gal H, Slabezki Y, Lensky Y. A preliminary report on the effect of origanum oil and thymol applications in honeybee (*Apis mellifera L.*) colonies in a subtropical climate on population levels of *Varroa jacobsoni*. Bee Sci. 1992;2 :(4) 175-180.

32. Ellis MD, Baxendale FP. Toxicity of seven monoterpenoids to tracheal mites (Acari: Tarsonemidae) and their honeybee (Hymenoptera: Apidae) hosts when applied as fumigants. J Econ Entomol. 1997;90 :(5) 1087-1091.

33. Oxfordshire Natural Beekeeping Group. Winter survival surveys show 'treatment-free' works [online] 2020: Available from: https://oxnatbees.wordpress.com/2020/08/05/winter-survival-surveys-show-treatment-free-works/.

34. Büchler R, Berg S, Le Conte Y. Breeding for resistance to *Varroa destructor* in Europe. Apidologie. 2010;41: (3) 393-408.

35. Kulhanek K, Garavito A, vanEngelsdorp D. Accelerated *Varroa destructor* population growth in honeybee (*Apis mellifera*) colonies is associated with visitation from non-natal bees. Sci Rep. 2021; 11**:** 7092.

36. Martin SJ. Naturally mite-resistant colonies evolve on Hawaii. Am. Bee J. 2020; 16: 649-651.

37. Thoms CA, Nelson KC, Kubas A, Steinhauer N, Wilson ME, vanEngelsdorp D. Beekeeper stewardship, colony loss, and *Varroa destructor* management. Ambio. 2019; 48: 1209-1218.

Formatted: Font: Italic

Formatted: Font: Italic
