# Supplementary material for: A survey of UK beekeeper’s Varroa treatment habits
Source: PLoS One. 2023 Feb 15;18(2):e0281130. doi: 10.1371/journal.pone.0281130 (PMC9931098; doi:10.1371/journal.pone.0281130)
Supplement: Attachment — Submitted filename: Reviewer detailed comments.docx [file pone.0281130.s003.docx]

Data is said to be publicly available, but no link included. Same goes for the supplemental information.

Please apply the use of commas for numbers consistently. For example, compare line 35 and line 38. Also *Varroa* is not consistently italicized.

I don’t understand why the feral population of bees is mentioned in line 43. You surveyed beekeepers with managed colonies. This has no implications for the feral bees and no evidence is presented that the UK has truly feral colonies.

Line 56-57, awkward sentence structure. Unclear what remained high.

Line 60, based used twice

Line 65-66, not clear why an IPM strategy would be linked to long-term resistance and low mite levels. IPM, as I understand it, is threshold base and involves monitoring and then treating appropriately. How does this encourage low mite levels? And why would this lead to long-term resistance? Needs explanation and evidence.

Line 102: I have no access to the Figure S1

Line 120: I have no access to Table S1

Line 201-203, that assumes that the survey reached and was completed by a representative wedge of the beekeeping population. Many treatment-free beekeepers tend to be younger and more active online, so this may have skewed the responses. These caveats should be mentioned.

Line 218-219: This is not surprising at all. Most treatment free beekeepers in the United States expect annual losses of 50%. Thus in order to rebuild the following year, they must keep more colonies in reserve. I am disappointed this survey didn’t ask for self-reporting of annual loss rates or honey production per colony. The high level of treatment-free suggests that beekeepers and the much higher colony numbers suggests that the resistance is probably not enough for low annual losses.

Line 235-240: This makes the assumption that IPM requires multiple treatments per year. IPM requires monitoring and treating accordingly when a pest level rises above a certain point. Also, many do not consider drone trapping a treatment. As I don’t have an actual copy of the survey questions, I can not assess if there may have been misunderstandings in the responses.

Line 253-255: This needs more clarification. Is the 4% what beekeepers reported was specifically due to varroa? This would then be an underrepresentation of actual winter losses due to varroa. Numerous surveys have shown that when varroa levels are high, winter losses increase, even though beekeepers blame other factors for the loss. Many beekeepers with colony winter losses don’t know what killed their colony, but beekeepers with more experience can often diagnose the contracted brood nest area and varroa frass.

Line 254-255: Why is the low winter loss attributed to treatment-free beekeeping. There could be other explanations, such as very active beekeeping groups that teach how to monitor and treat effectively. The evidence presented does not warrant this statement, as there is no segregation of losses by those treating and those not treating presented.

Line 256-260: These statements require the reader to go to the original articles and assess the numbers of colonies surveyed. Important information such as colonies assessed in the survey should be included with the paraphrased results, especially as these are not peer-reviewed studies but association results and so the statistics have not been evaluated. The way they are presented in this paper, the reader assumes they are peer-reviewed papers, unless the references are checked in detail.

Line 276-279: This is a very broad statement and is not justified by the results presented, as no losses were calculated in the survey.
